# Parkinson’s Critical Heart Rate Test: Applying the Critical Power Model for People with Parkinson’s Disease

**DOI:** 10.5114/jhk/186562

**Published:** 2024-05-17

**Authors:** Ricardo Augusto Barbieri, Carlos Augusto Kalva-Filho, Murilo Henrique Faria, Aline Prieto Silveira-Ciola, Camila Torriani-Pasin, Lucas Simieli, Fabio Augusto Barbieri

**Affiliations:** 1 Department for Life Quality Studies, University of Bologna (UNIBO), Rimini, Italy.; 2 School of Sciences, Department of Physical Education, Human Movement Research Laboratory (MOVI-LAB), São Paulo State University (Unesp), Bauru, SP, Brazil.; 3 Department of Physical Therapy and Movement Sciences, University of Texas, El Paso, USA.

**Keywords:** validity, exercise intensity, aerobic exercise, testing, reliability

## Abstract

Aerobic exercise with the correct intensity can attenuate motor and non-motor symptoms of Parkinson's disease (PD) and improve the quality of life. However, a specific, validated, non-invasive, and outside the laboratory protocol that assesses physiological variables to prescribe optimal aerobic exercise intensity for people with PD is nonexistent. Therefore, this study aimed to propose a protocol, the Parkinson's critical heart rate test (Parkinson-CHR test), to determine the critical heart rate (CHR) in individuals with PD and verify its validity, reliability, and sensitivity. Fifteen people with idiopathic PD, who were able to practice exercises, were recruited to participate in the study (71.1 ± 6.6 years). The study consisted of two experiments: i) the first one aimed to assess the validity and reliability of the protocol, with participants performing the test twice at a one-week interval; ii) the second experiment aimed to investigate the protocol sensitivity, with individuals being evaluated before and after an 8-week training program according to Parkinson-CHR intensity. In experiment 1, no differences between test and retest were observed in the time to cover the distances (400, 800 and 1200 m), the total heart rate, the critical heart rate, and critical speed (p > 0.05). In experiment 2, there was a reduction in time to cover 400 and 800 m as well as in the total heart rate for all distances after the 8-week training program. The Parkinson-CHR test is a reliable, reproducible, inexpensive, and non-invasive protocol to assess, prescribe, and monitor aerobic exercise intensity in people with PD.

## Introduction

Aerobic exercise has beneficial effects on improving balance and gait in people with Parkinson’s disease (PD) (Zhen et al., 2022). However, the favorable effects of aerobic exercise are dependent on the intensity of the exercise. High or vigorous aerobic exercise intensity can lead to greater adaptation to brain neuroplasticity (Fernandes et al., 2020; Fisher et al., 2008; Kelly et al., 2017) and promote mobility improvements (Miller Koop et al., 2019) compared to low-intensity aerobic exercise in people with PD. Thus, determining and monitoring aerobic exercise intensity is key for the efficiency of exercise prescription and treatment in people with PD, stimulating positive adaptation and maximizing benefits. However, the number of protocols that assess physiological variables to prescribe the optimal exercise intensity for people with PD is limited.

The intensity of the peak oxygen uptake (VO_2peak_) during a maximal incremental effort (i.e., maximum cardiorespiratory stress) is often used to prescribe and monitor aerobic exercise. Katzel et al. (2011) demonstrated that the measurement of VO_2peak_ was reliable and repeatable in people with PD. However, VO_2peak_ assessment is dependent on expensive equipment to be used under laboratory conditions and with supervision of qualified personnel. Also, there are two other limitations of this protocol: (a) testing is performed on a treadmill, which can guide the walking of people with PD, what may interfere with the evaluation (Warlop et al., 2018); and (b) the intensity correspondent to the peak oxygen uptake is reached in the severe intensity domain, in which the time to exhaustion is finite and fatigue is observed rapidly (Hill et al., 2002), which may be a challenge for people with PD (Katzel et al., 2011). Thus, to determine submaximal markers of intensity, specifically the boundary between heavy and severe domains (i.e., anaerobic threshold), it is useful to prescribe a tolerable exercise session for people with PD. In addition, to the best of our knowledge, there is currently no specific, validated, non-invasive, and outside-of-laboratory protocol available to determine the appropriate aerobic exercise intensity for people with PD. Most training or rehabilitation interventions for people with PD rely on protocols that were originally designed and validated for healthy individuals, primarily older adults, to assess aerobic exercise intensity.

An alternative to determine the heavy-severe boundary—with high levels of practicality and low costs—is the critical power model (Bergstrom et al., 2015; Hill et al., 2002; Mielke et al., 2011). In this model, the relationship between distance and time is determined by measuring the time to complete several distances. The heavy-severe boundary is determined by the slope of the resulting distance-time relationship (i.e., critical speed) (Hill et al., 2002). The exercise prescribed below the critical speed can be continuous and tolerable for a long time, while the exercise intensity at the critical speed could be prescribed continuously for a shorter time (30 to 60 min), and the intensities above the critical speed would be prescribed only through intermittent exercise approaches. Besides the critical speed, an external marker of intensity, the same protocol allows for determining the heart rate (HR) corresponding to the moderate-severe boundary (i.e., critical HR), which represents an internal marker of intensity (Bergstrom et al., 2015; Mielke et al., 2011).

Despite the high applicability of the critical power model for aerobic exercise prescription in older adults (Colucci et al., 2020), no studies have tested the reproducibility and sensibility of this model in people with PD, limiting its use to assess, prescribe, and monitor training adaptations in PD. Therefore, the purpose of this study was i) to investigate the reliability and test-retest reproducibility of the critical speed and HR models in people with PD, validating the Parkinson's Critical Heart Rate Test (Parkinson-CHR test), and ii) to verify the response (sensibility) of the Parkinson-CHR test after an 8-week aerobic training program according to CHR intensity.

## Methods

### Participants

Fifteen people with idiopathic PD were selected from a specialized Parkinson’s disease center in Bauru, Brazil (Ativa Parkinson – UNESP) to participate in the study (Table 1). The study was approved by the research ethics committee of the São Paulo State University (Unesp) (approval code: CAAE 17892819.9.0000.5398; approval date: 12 September 2019) and each participant provided written informed consent.

**Table 1:** Characteristics of participants with PD.

	Age (years)	Height (m)	Body mass (kg)	UPDRS II (pts)	UPDRS III (pts)	H&Y (pts)	MMSE (pts)	TMT A (s)	TMT B (s)	TUG (s)
1 (M)*	64	1.69	74.0	8	25	2	29	37.72	73.22	5.22
2 (M)	76	1.65	58.0	14	31	2	27	82.60	146.08	7.03
3 (F)	78	1.45	44.4	7	23	2	27	53.35	210.05	6.88
4 (M)*	68	1.65	72.0	12	48	2	24	67.84	153.29	4.96
5 (F)*	67	1.57	69.8	16	33	2	26	37.21	143.98	6.22
6 (F)	77	1.40	48.2	13	52	2.5	21	227.64	153.29	7.60
7 (M)	83	1.61	70.0	6	20	2	30	76.63	173.19	7.37
8 (F)*	78	1.46	48.8	10	24	2	28	22.92	205.67	8.27
9 (M)	72	1.60	65.0	10	39	2.5	26	67.84	153.29	11.36
10 (M)*	82	1.67	81.6	13	31	2	28	79.64	155.09	6.39
11 (M)	67	1.74	97.0	14	33	2	30	45.00	109.94	6.12
12 (F)*	66	1.58	49.0	9	27	2.5	25	27.22	61.91	5.42
13 (M)*	61	1.74	87.0	6	20	1.5	29	66.29	168.38	7.30
14 (F)	66	1.58	49.0	8	29	2	29	76.00	153.29	5.81
15 (M)*	74	1.67	86.0	14	24	2	27	49.76	238.69	7.57
**Experiment 1 (n = 15)**										
**Average**	**71.93**	**1.60**	**66.6**	**10.6**	**30.6**	**2.1**	**27.1**	**67.84**	**153.29**	**6.90**
**SD**	**6.87**	**0.10**	**16.6**	**3.2**	**9.4**	**0.3**	**2.4**	**48.26**	**46.80**	**1.57**
**Experiment 2 (n = 8)**										
**Average**	**70.00**	**1.63**	**71.03**	**11.00**	**29.00**	**2**	**27.00**	**45.82**	**149.56**	**6.42**
**SD**	**7.27**	**0.09**	**15.04**	**3.34**	**8.72**	**0.3**	**1.85**	**20.75**	**59.80**	**1.20**

* Participants included in the experiment 2. M: male; F: female; UPDRS: Unified Parkinson’s Disease Rating Scale; H&Y: Hoehn and Yahr Scale; MMSE: Mini-Mental State Exam; TMT: Trail Making Test (Part A and B); 6MWT: six-minute walk test.

Only individuals at stages I to III in the Hoehn and Yahr Scale (H&Y) were included in the study. The diagnosis of PD, assessed for more than six months, was confirmed by expert neurologists following the UK Parkinson’s Disease Brain Bank criteria (Hughes et al., 1992). Participants should be under dopaminergic medication therapy, without changes in medication therapy during the previous three months, and able to walk independently. Throughout the study period, participants were instructed to maintain their drug treatment routine (periods and doses). Participants were evaluated and trained in the ON-state of dopaminergic medication (approximately one hour after taking the medication) (Araújo-Silva et al., 2022). For both experiments, participants with (1) other parkinsonism syndromes and/or neurological diseases, (2) uncontrolled cardiovascular, metabolic, and/or inflammatory diseases, (3) cardiovascular contraindications to exercise, (4) rheumatic and/or orthopedic diseases that impaired exercise participation in high-intensity exercise, (5) asthma and/or chronic obstructive pulmonary disease, and/or (6) exhibiting signs of cognitive decline (i.e., Mini-Mental State Exam) (Folstein et al., 1975) were excluded.

### Design and Procedures

**Figure 1:**
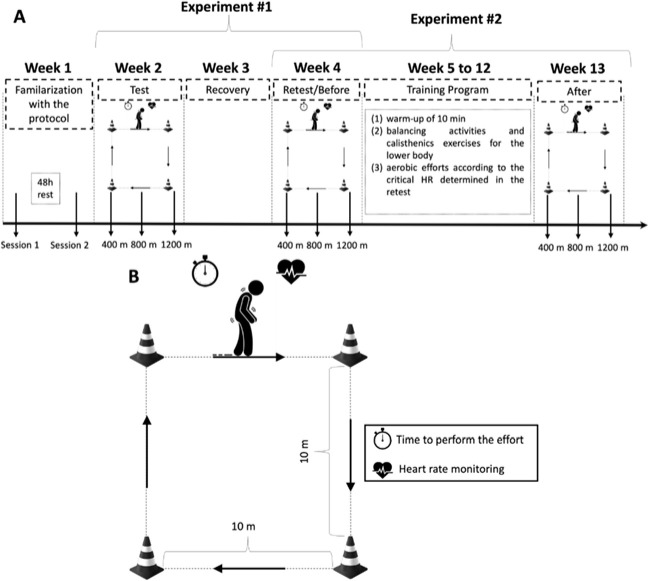
A: Experimental design of the study; B: square circuit of the protocol test.

Two experiments were run to attend to the purposes of the study (Figure 1A). Seven days before experiment 1, participants were familiarized with the procedures, performing a simulated protocol in two different sessions separated by 48 h, and clinical outcomes were obtained. The daily activities and motor portion of the Unified Parkinson’s Disease Rating Scale (UPDRS-II and III), H&Y, Mini-Mental State Exam, Trail Making Test (TMT), and timed up-and-go test (TUG) were collected to characterize the sample. In experiment 1, participants (n = 15) underwent three efforts of 400, 800, and 1200 m (test) in random order, with an interval of 48 h between subsequent efforts. After seven days of recovery, i.e., no structured/effortful physical activity, the three distances were repeated (retest). The time to perform walking and the HR in each effort were monitored. In experiment 2, participants (n = 8) completed an 8-week aerobic exercise program according to CHR intensity. They were assessed before and after the intervention through the Parkinson-CHR test. In addition, to confirm the efficacy of the training program, the six-minute walk test (6MWT) (ATS Committee, 2002) was performed before and after the intervention. The 6MWT is a submaximal test commonly applied to assess aerobic fitness of people with PD (Falvo and Earhart, 2009). Only participants who attended at least 90% of the training sessions were included in further analyses.

#### Critical Speed and HR Models (Parkinson-CHR test)

The concepts of critical power adapted from Bergstrom et al. (2015) were used. Three randomly ordered efforts (400 m, 800 m, and 1200 m) were performed in a time duration range (Bergstrom et al., 2015; Jones and Vanhatalo, 2017). Participants were instructed to travel the distance as fast as possible with no running allowed in a square circuit of 10 x 10 m (Figure 1B) in an indoor environment, with a rigid surface. The total time to complete the distance was recorded (Vollo® Sports Stopwatch; 0.01 s approach, Cotia, Brazil) and the HR was continuously monitored and recorded as 5-s averages (1 Hz; Polar Team - Polar® - Kempele, Finland) during the test (Figure 1).

**Figure 2:**
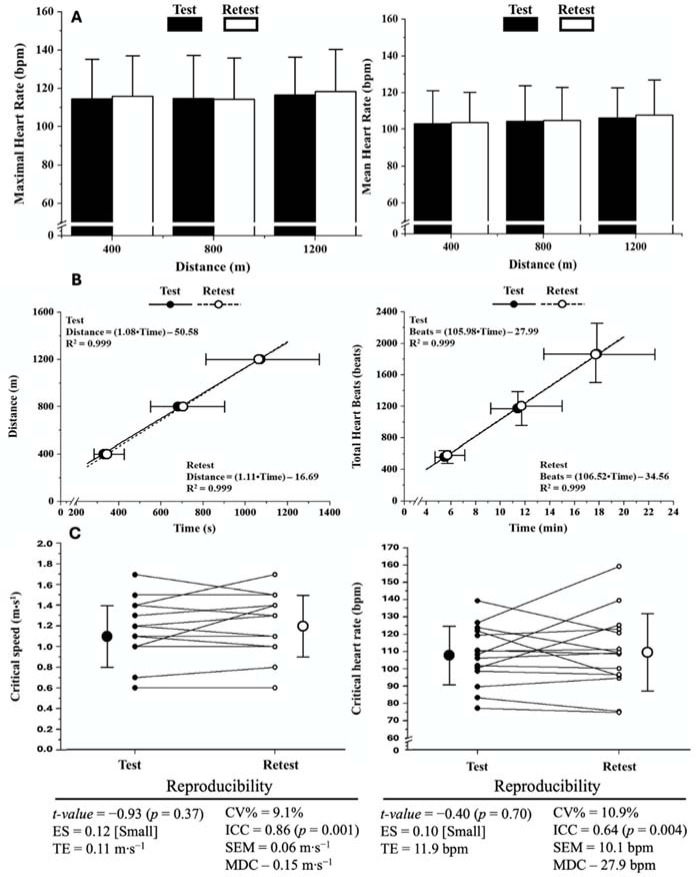
A: HR responses under the different effort conditions during test and retest; B: Critical speed (left) and critical HR (right) models constructed based on test and retest. The equations were obtained through the mean values of the time spent per distance covered (critical speed model) or the total heartbeats per time spent under each effort condition (critical HR model); C: Critical speed and critical HR models obtained based on test and retest. The scatter plots connected by lines represent the individual data. ES: effect size, TE: typical error; CV%: coefficient of variation; ICC: intraclass correlation coefficient; SEM: standard error of measurement; MDC: minimal detectable change.

The total number of heartbeats (HBs) was calculated as the product of the average 5-s HR (b·min^−1^) and the time to complete the distance. The HB plotted for each speed as a function of the time spent at each distance. The CHR was defined as the slope coefficient of the regression line between the HB and time. The critical speed was the slope of the distance-time relationship (Jones and Vanhatalo, 2017).

#### Training Program

The training program lasted eight weeks, twice per week (approximately 50 min per day) with a minimum interval of 48 h between sessions. The training session was composed of three parts: (1) a warm-up of 10 min with stretching and mobility activities; (2) balancing activities and strength exercises (i.e., calisthenics exercises) for the lower body using body weight. The duration of the second part was reduced according to the third part increased duration (15 min from the 1^st^ to the 4^th^ week and 10 min from the 5^th^ to the 8^th^ week); (3) aerobic exercise (walking) according to the critical HR determined in the retest of the first experiment. The progression of the third part was done by increasing its duration during the eight weeks of the intervention: 20 min from the 1^st^ to the 4^th^ week, and 30 min from the 5^th^ to the 8^th^ week. Participants performed walking in a rectangular circuit with obstacles on the floor (e.g., cones, steps, and barriers) to increase their engagement in the activity. The HR was continuously monitored during training sessions. Participants were instructed to keep their HR above the critical HR, receiving constant feedback about it. When participants could not keep the HR above the critical value, they were encouraged to do the exercise as fast as they could.

### Statistical Analysis

Statistical analysis was performed using SPSS® Statistics software, version 20.0 (IBM Corporation®) with the level of significance set at *p* < 0.05. The data normality was confirmed through the Shapiro-Wilk test, allowing its description using the mean and standard deviation. To compare the characteristics of participants between groups of experiments 1 and 2, the Student’s *t*-test for independent samples was applied. For experiment 1, test-retest reproducibility was investigated using the Student’s *t*-test for dependent samples, typical error (TE), the coefficient of variation (CV%), and the intraclass correlation (ICC) (Hopkins, 2000). Paired comparisons were accompanied by Cohen’s *d* [effect size (ES)], which was interpreted as small (≤0.20); moderate (≤0.50); large (≤0.80), or very large (>0.80). In addition, the standard error of measurement (SEM = SD · √(1 − ICC)) and the minimum detectable change at a 95% confidence level (MDC = SEM · 1.96 · √2) were assessed (Portney et al., 2015). A mixed model analysis using fixed factors (i.e., ‘condition’ and ‘HR during efforts’) and a repeated and random factor (i.e., subject) was used to test whether the HR response throughout the effort conditions was different between the test and retest conditions followed by the Sidak’s post-hoc test, when necessary. For experiment 2, HR responses during the effort conditions were tested through the mixed model analysis described in the first experiment. In addition, training effects on critical speed, the HR, and the 6MWT were calculated using the Student’s *t*-test for repeated samples, accompanied by the ES values. In our analyses, we only included participants who performed all the training sessions in this experiment.

## Results

The characteristics of participants in experiments 1 and 2 are shown in Table 1. Note that participants of experiment 2 are marked with an asterisk (n = 8).

### Experiment 1

Figure 2A demonstrates HR responses during test-retest conditions. The maximal and mean HR values were similar across distances (*p* > 0.91) and between test-retest conditions (*p* > 0.71). No differences between test-retest conditions were observed for the time to complete 400 m (*p* = 0.24; ES = 0.30 [Moderate]), 800 m (*p* = 0.36; ES = 0.14 [Moderate]) and 1200 m (*p* = 0.79; ES = 0.03 [Small]). In addition, the total number of HBs was similar between test-retest for all conditions (400 m: *p* = 0.17; ES = 0.26 [Moderate]; 800 m: *p* = 0.52; ES = 0.15 [Moderate]; 1200 m: *p* = 0.88; ES = 0.02 [Small]).

The critical speed and HR models constructed based on the test-retest conditions are presented in Figure 2B. High levels of linearity were observed for critical speed (coefficient of determination R^2^: test > 0.98; retest > 0.97), and HR models (coefficient of determination R^2^: test > 0.96; retest > 0.90). The reproducibility of the critical speed and critical HR are presented in Figure 2C. No significant differences were observed between test-retest conditions. Finally, low values of TE, CV%, SEM, and MDC were accompanied by significant ICC values (Figure 2C).

### Experiment 2

**Figure 3:**
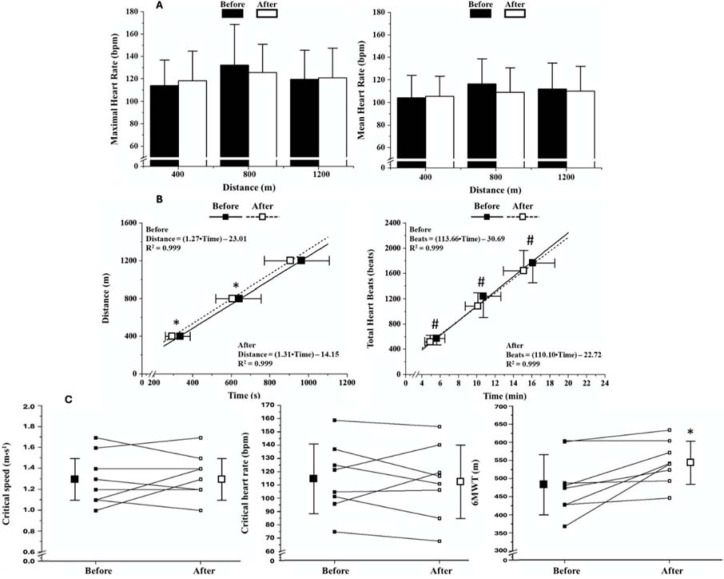
A: HR responses during the effort conditions performed before and after aerobic training; B: Critical speed (above) and critical HR (below) models observed before and after the training period. The equations were obtained through the mean values of the time spent per distance covered (critical speed model) or the total heartbeats per time spent under each effort condition (critical HR model); C: Training effects on critical speed, critical HR, and distance covered during the six-minute walking test (6MWT). The scatter plots connected by lines represent individual data. * significant differences after training for time spent during the effort condition; # significant differences after training for total heartbeats during the effort condition; $ significant differences from baseline values.

Of the 15 participants, only 8 participants attended 90% of the training sessions and were included in the analysis (Table 1). The HR during the training program was on average 69.5 ± 13.2% of the maximal HR values predicted for the individual’s age (157.2 ± 4.7 bpm). HR responses across effort conditions in the Parkinson-CHR test before and after the training period (*p* > 0.48) were not altered (*p* > 0.39) (Figure 3A). Time spent to complete 400 m (*p* = 0.003; ES = 0.80 [Large]) and 800 m (*p* = 0.05; ES = 0.31 [Moderate]) decreased after the intervention, while no significant changes were observed for 1200 m (*p* = 0.12; ES = 0.39 [Large]) (Figure 3B). The total HR decreased after training under all conditions (400 m: *p* = 0.01; ES = 0.54 [Large]; 800 m: *p* = 0.05; ES = 0.55 [Large]; 1200 m: *p* = 0.02; ES = 0.39 [Moderate]) (Figure 3B).

Figure 3B shows the critical speed and critical HR model before and after training. Trivial changes were observed for critical speed (*p* = 0.44; ES = 0.16 [Small]) and critical HR (*p* = 0.69; ES = 0.08 [Small]) (Figure 3C). The 6MWT results (Figure 3C) showed improvements after the intervention (*p* = 0.02; ES = 0.84 [Large]).

## Discussion

We verified the reliability and reproducibility of a simple, easily measured, and practical protocol (Parkinson-CHR test) to determine the exercise intensity using critical speed and HR models in people with PD. Our results showed that the Parkinson-CHR test was reliable with high levels of reproducibility. Also, we investigated the response (sensibility) of the Parkinson-CHR test after an 8-week aerobic training program. Despite no changes in critical speed and the CHR after training, the time to cover 400 and 800 m, and the total HR in the distances of 400, 800, and 1200 were significantly lower compared to before the training program, representing an adaptation (sensibility) to the eight-week training program. Also, the typical error, the coefficient of variation, and the minimum detectable change at a 95% confidence level showed a lower level.

Thus, the sensibility of the Parkinson-CHR test was confirmed, indicating that the Parkinson-CHR test may have practical applicability to monitoring aerobic training adaptations. Our findings advanced the knowledge of aerobic assessment in people with PD and introduced a new non-invasive low-cost and easy-to-apply tool.

### Reliability and Reproducibility of the Parkinson-CHR Test

The critical power concept was proposed initially by Monod and Scherrer (1965) to determine the amount of work performed at exhaustion for individual and synergistic muscle groups and estimate the anaerobic work capacity. This model has been widely applied in different sports with a focus on intermittent high-intensity exercise (Jones and Vanhatalo, 2017). Our results demonstrated that the effort conditions applied to determine critical speed were well tolerated by people with PD at the mild to moderate disease stage, and showed high levels of reproducibility, demonstrating its applicability to assess different levels of aerobic fitness (i.e., high ICC values) and prescribe aerobic exercise (i.e., low levels of TE, CV%, SEM, and MDC) in people with PD.

The critical HR (Mielke et al., 2011), which can represent a sustainable intensity (30 to 60 min) (Bergstrom et al., 2015), was tested during arm-ergometer exercise in older individuals, and the average HR measured in the CP test was similar to the critical HR calculated by a mathematical model (Mielke et al., 2011) with 139.7 ± 13.3 bpm and the intensity of 66.8 ± 9.4% of the peak work rate (Colucci et al., 2020). These results were similar to the findings of the present study, in which the maximal critical HR was 69.5 ± 13.2% of the maximal values of the age-predicted maximal HR (157.2 ± 4.7 bpm). In addition, our results showed a low value of typical error, the coefficient of variation, standard error of measurement, and the minimum detectable change at a 95% confidence level, which indicated slight differences between test-retest conditions and, consequently, that critical HR appeared to be sensible to training adaptations. However, ICC values were moderate, indicating that the use of the CHR to discriminate between different levels of aerobic fitness may be limited.

### Sensibility of the Parkinson-CHR Test

To determine the sensibility of the test, it is necessary to present the smallest worthwhile effect that can be detected or compare the responses before and after the exercise intervention (Currell and Jeukendrup, 2008). Our findings showed a low value of typical error, the coefficient of variation, standard error of measurement, and the minimum detectable change at a 95% confidence level. In addition, the Parkinson-CHR test was able to identify improvement in aerobic fitness in people with PD, showing a reduction in time to cover 400 and 800 m and the total HR for all distances. Furthermore, people with PD from the present study increased the distance covered during the 6MWT, confirming the adaptations from the aerobic training program. These findings agree with other studies available in the literature (Arfa-Fatollahkhani et al., 2020; Atan et al., 2019) which showed that aerobic training improves performance of people with PD in the 6MWT. Considering the adaptations after aerobic training, we can consider that the Parkinson-CHR test was also sensitive to monitoring aerobic training in people with PD. On the other hand, no significant changes in the critical speed after the 8-week aerobic training program were found.

The lack of significant change in the critical speed after the training program may be explained by the lack of reduction in time to cover 1200 m after the intervention. We found a significant reduction in time to cover both 400 and 800 m, but not in 1200 m. It was suggested in the proposed critical HR that subjects should complete efforts in approximately 8 to 20 min before exhaustion (Mielke et al., 2011). However, Monod and Scherrer (1965) indicated that the longest duration of the effort should be 12–15 min in maximum. In the present study, time to complete 1200 m was around 16 ± 2.4 min at baseline and 15 ± 2.2 min after the training intervention, which were higher values than recommended. The longest duration to complete 1200 m may affect the mathematic equation to determine the critical speed. In addition, people with PD have higher sensibility of fatigue (Siciliano et al., 2018) and reach exhaustion earlier than their peers (Finsterer and Mahjoub, 2014). Thus, the longest distance during the test can overestimate aerobic fitness of this population and reduce the sensibility of the test after aerobic training. Therefore, one may argue that a distance possible to be covered in a shorter time (e.g., 12–15 min) could be more sensitive to detect adaptations in critical speed. Thus, we suggest that future studies test the sensibility of the Parkinson-CHR test with reduced distance to be covered such as 300, 600, and 900 m. Furthermore, although aerobic training leads to an improved adaptation of endothelial reactivity and aerobic capacity in people with PD (Fernandes et al., 2020), one may argue that the critical HR maintains the same HR for relative intensity, without changing the critical HR considering the same target intensity even with the improvement of aerobic fitness (including the total HR) after aerobic training. It is important to highlight that this is the first study to verify the sensibility of the critical HR to training effects, consequently, more studies will be needed for a better understanding of critical heart responses.

A final consideration about the absence of change in critical speed and the HR is necessary. We prescribed an 8-week aerobic training program for people with PD, which may not be long enough to cause adaptations in critical speed and the HR. Mak et al. (2017) recommended at least 12 weeks to produce long-term benefits of aerobic training. The authors indicated that changes in aerobic fitness robust variables, such as motor-learning-related brain changes (Duchesne et al., 2016), require more training time to adapt. Perhaps critical speed and the HR are also robust variables, demanding more training time for a significant positive adaptation. Thus, it is hypothesized that longer aerobic training (more than 12 weeks) may significantly improve critical speed and the HR, and, consequently, the sensibility of the Parkinson-CHR test.

### Limitations and Practical Recommendations

Critical speed and the HR are indirect methods for determining aerobic fitness, thus, future studies should test the correlations between the CHR and other indexes of aerobic fitness (e.g., peak oxygen uptake and the anaerobic threshold). Another limitation of the present study was the use of the effort of 1200 m, in which the average duration to cover this distance was higher than recommended (12–15 min). Besides, one can consider a small sample size in experiment 2, even though statistical analyses showed moderate to large statistical power in training-induced improvements. Also, the validation, reproducibility, and sensibility of the Parkinson-CHR test were only evaluated in people with PD in a moderate level of severity (H&Y < 3) and with no freezing of gait, indicating that tolerance of the proposed distances should be tested in more advanced people. In addition, the tolerance of people with PD for a square-wave test using the critical speed for intensity prescription remains unclear, consequently, future studies would provide valuable information about the real applicability of this variable and the expected physiological responses (e.g., below, at or above the CHR and CS).

The main practical recommendations to use the Parkinson-CHR test are: i) to perform comprehensive familiarization, considering that the test involves changes of direction, which may be difficult for people with PD and interfere with test results, ii) to keep the duration of efforts between 3 and 12 min, iii) to consider the characteristics of participants (sedentary, moderately active or active) and the level of disease before testing, iv) no need of advanced equipment (e.g., VO_2_ mask or sophisticated software), v) to use the test to prescribe the external (critical velocity) and internal (critical HR) workload of training programs, vi) to have an evaluator close to the participant (back) for possible imbalance or freezing episodes, as it is a circuit including changes in direction, vii) to choose a place for test application with a smooth floor and without slopes or irregularities, viii) not to give any verbal stimulus to the participant during the test to avoid interference in the performance; also, all verbal instructions must be explained before the start of the test, ix) to be aware of participants with low scores in the Mini-mental test, as they may have difficulties in understanding verbal instructions, which may impact the test performance, and x) not to run during the test to ensure safety.

## Conclusions

In conclusion, the Parkinson-CHR test is a valid, reliable, sensitive, low-cost, safe, and non-invasive method to estimate aerobic exercise intensity, as well as to prescribe and monitor aerobic training intensity in people with PD. Also, we can conclude that the Parkinson-CHR test is adequate to prescribe a tolerable exercise session for people with PD.
